# The role of fibrinolysis inhibition in engineered vascular networks derived from endothelial cells and adipose-derived stem cells

**DOI:** 10.1186/s13287-017-0764-2

**Published:** 2018-02-12

**Authors:** Severin Mühleder, Karoline Pill, Mira Schaupper, Krystyna Labuda, Eleni Priglinger, Pablo Hofbauer, Verena Charwat, Uwe Marx, Heinz Redl, Wolfgang Holnthoner

**Affiliations:** 1 Ludwig Boltzmann Institute for Experimental and Clinical Traumatology, AUVA Research Centre, Donaueschingenstrasse 13, A-1200 Vienna, Austria; 2 Austrian Cluster for Tissue Regeneration, Vienna, Austria; 3 Institute of Molecular Biotechnology of the Austrian Academy of Science (IMBA), Vienna Biocenter (VBC), Vienna, Austria; 4 Department of Biotechnology, University of Natural Resources and Life Sciences (BOKU), Vienna, Austria; 5 TissUse GmbH, Berlin, Germany; 6 Present address: Division of Plastic and Reconstructive Surgery, Department of Surgery, Medical University of Vienna, Vienna, Austria

**Keywords:** Vascularisation, Fibrin, Endothelial cells, Adipose-derived stem cells

## Abstract

**Background:**

Co-cultures of endothelial cells with mesenchymal stem cells currently represent one of the most promising approaches in providing oxygen and nutrient supply for microvascular tissue engineering. Still, to translate this model into clinics several in vitro parameters including growth medium and scaffold degradation need to be fine-tuned.

**Methods:**

We recently described the co-culture of adipose-derived stem cells with endothelial cells in fibrin, resulting in capillary formation in vitro as well as their perfusion in vivo. Here, we aimed to further characterise microvascular tube formation in fibrin by determining the role of scaffold degradation, thrombin concentration and culture conditions on vascularisation.

**Results:**

We observed that inhibition of cell-mediated fibrin degradation by the commonly used inhibitor aprotinin resulted in impaired vascular network formation. Aprotinin had no effect on laminin and collagen type IV deposition or formation of tube-like structures in scaffold-free co-culture, indicating that poor vascularisation of fibrin clots is primarily caused by inhibition of plasminogen-driven fibrinolysis. Co-culture in plasminogen- and factor XIII-depleted fibrin did not result in different vascular network density compared to controls. Furthermore, we demonstrate that thrombin negatively affects vascular network density at high concentrations. However, only transient activation of incorporated endothelial cells by thrombin could be observed, thus excluding a long-term inflammatory response in tissue-engineered micro-capillaries. Finally, we show that vascularisation of fibrin scaffolds in basal medium is undermined because of increased fibrinolytic activity leading to scaffold destabilisation without aprotinin.

**Conclusions:**

Taken together, our data reveal a critical role of fibrinolysis inhibition in in vitro cell-mediated vascularisation of fibrin scaffolds.

**Electronic supplementary material:**

The online version of this article (10.1186/s13287-017-0764-2) contains supplementary material, which is available to authorized users.

## Background

The integration of a perfusable microvascular system into tissue-engineered constructs still constitutes one of the main challenges in the creation of artificial tissues [1]. Besides several technical approaches like three-dimensional (3D)-printing, 2-photon-polymerisation [2] or the de- and recellularization of vascular trees [3], mainly co-cultures of endothelial cells (EC) with supporting cells have been investigated [4]. For instance, fibroblasts support the formation of vascular networks when co-embedded with EC into a fibrin hydrogel [5]. In addition, osteoblasts incorporated in silk-fibroin matrices have been reported to induce ingrowth of endothelial cells and vascularisation of implanted scaffolds in vivo [6]. We recently established a co-culture system by integrating adipose-derived stromal/stem cells (ASC) with endothelial colony-forming cells (ECFC) in a fibrin matrix [7]. This system provides autologous features since both ASC and ECFC can potentially be harvested from the same individual. Furthermore, fetal calf serum (FCS) can be substituted with human platelet lysate, thus promoting the autologous character of this system [8]. Angiogenic stimuli are expressed by ASC, however, also the close contact of both cell types is required for microvascular tube formation [9]. Scaffold design is crucial to promote cell growth and regeneration. In general, the scaffold used should match the mechanical properties of the target tissue and its rate of degradation should be fine-tuned with the respective tissue growth rate [10]. In this context, fibrin is a suitable material for soft tissue engineering, since its mechanical properties match the target tissue, and its concentration and the degradation rate can be fine-tuned by fibrinogen and thrombin concentrations [11]. While several studies report the formation of vascular networks in fibrin [12, 13], we still do lack understanding of many critical factors involved in vascular tube formation. Importantly, the influences of cell culture parameters need to be considered in order to optimise this complex process to translate such methods into clinics. Ideally, vascularised scaffolds should be maintained in chemically defined medium that is free of any (xenogenic) serum components to enable the use of such scaffolds in patients [8, 14, 15].

Inhibition of fibrinolysis by blocking plasmin formation is frequently used for vascularisation in tissue engineering to control the degradation rate in vivo and in vitro [16–19]. For this purpose, the serine protease inhibitor aprotinin is commonly used to antagonise plasminogen activation [17, 20]. Other means to control scaffold degradation and stability include mixing fibrin with another material such as collagen [20, 21]. Interestingly, it has been shown that the use of aprotinin reduces VEGF-induced capillary-like structure formation of endothelial cells in fibrin hydrogels [20]. Still, we and others have used aprotinin to promote scaffold stabilisation in fibrin co-culture of endothelial cells with supporting cell types such as fibroblasts or ASCs [9, 19]. Additionally, many more studies exist where aprotinin was employed to promote acellular scaffold stability upon implantation [17, 19, 22]. We therefore aimed to revise the current understanding of aprotinin in microvascular tissue engineering applications. We measured fibrinolysis by using fluorescence-labelled fibrinogen in an EC-ASC co-culture system and showed that inhibition of fibrinolysis by aprotinin correlated with a decrease in vessel density and vessel diameter. We furthermore show the necessity of aprotinin in serum- and growth factor-free culture and characterised laminin and collagen type IV production by newly formed microvasculature structures.

## Methods

### Cell culture

ASC were isolated from liposuction material as described before [23] and used in passages 2–10. Pooled donor human umbilical vein endothelial cells (HUVEC) were purchased from Lonza (Walkersville, MD, USA) and used in passages 5–13. GFP-HUVEC used for live-cell imaging were purchased from Olaf Pharmaceuticals (Worcester, MA, USA) and used in between passage 5 and 8. All cells were maintained in endothelial growth medium-2 (EGM-2) that consists of endothelial basal medium-2 (EBM-2) (Lonza, Walkersville, MD, USA,, termed “basal medium” throughout the manuscript) supplemented with 2% FCS, hydrocortisone, fibroblast growth factor-2 (FGF-2), vascular endothelial growth factor (VEGF), R3-insulin-like growth factor-1 (R3-IGF-1), ascorbic acid, epidermal growth factor (EGF), GA-1000 and heparin (EGM-2 SingleQuots, Lonza, Walkersville, MA, USA) and additional FCS (Sigma-Aldrich Co. LLC., St. Louis, MO, USA to a final concentration of 5%) was added if not stated otherwise. This medium is termed “full medium” throughout the manuscript. ECFC were isolated from healthy donors and characterised as described previously [7]. Briefly, 30 ml of peripheral blood were used to isolate peripheral blood mononuclear cells via centrifugation with lymphocyte separation medium (LSM 1077, GE Life Sciences, Chalfont St Giles, UK). Cells were then resuspended in full medium and seeded onto collagen type I/III-coated six-well plates. First EC colonies with cobblestone morphology were visible after 1–2 weeks. ECFC were cultured on fibronectin-coated plastic (2 mg/ml, fibronectin from human plasma, Sigma-Aldrich Co. LLC., St. Louis, MO, USA) in full medium and used in passage 3.

### Plasmids and retroviral infection of HUVEC

EGFP and mCherry in pLV vectors and pBMN-Z were purchased from Addgene (Cambridge, MA, USA). pcDNA3-EYFP-HIS was purchased from Invitrogen (ThermoFisher, Waltham, MA, USA). EGFP and mCherry were subcloned into pBMN after digestion with *Bam*HI and *Sal*I. EYFP-HIS was subcloned into pBMN after digestion with *Bam*HI and *Eco*RI. Phoenix ampho cells were a kind gift of Regina Grillari (University of Natural Resources and Life Sciences, Vienna, Austria) and cultured in DMEM 10% FCS. Virus particle generation was performed by transfecting Phoenix ampho cells at 80% confluency using lipofectamine 2000 or TurboFect (Thermo Fisher, Waltham, MA, USA) according to the manufacturer’s instructions. Supernatant containing virus particles was mixed 50:50 with full medium and transferred onto 80% confluent HUVEC and incubated overnight. YFP-HUVEC, GFP-HUVEC and mCherry-HUVEC were then expanded in new flasks and used for subsequent experiments. The label was chosen based on the prerequisites of the respective experiment (e.g. complementary stainings with fluorescent dyes).

### Embedding of cells in fibrin matrices

Fibrin matrices were generated as described previously [9, 15]. Briefly, fibrin gel components (TISSEEL®, Baxter, Vienna, Austria) were prepared by warming frozen fibrinogen to room temperature and diluting 4 U/ml thrombin 1:10 in calcium chloride (CaCl_2_). Where indicated, we tested another fibrinogen product that was free of plasminogen, factor XIII (FXIII) and fibronectin (fibrinogen peak 1, FP1, CoaChrom, Maria Enzersdorf, Austria). All fibrinogen components were prepared without addition of aprotinin to exclude any potential influence. Cells, fibrinogen and thrombin were mixed and pipetted onto prepared coverslips. Gels polymerised at room temperature for 30 min before growth medium with or without aprotinin was added. The final concentration of fibrinogen used was 2.5 mg/ml, of thrombin 0.2 U/ml and of aprotinin 100 KIU/ml if not stated otherwise. Fibrin scaffolds had a volume of 200 μl and contained 500 endothelial cells and 500 ASC per μl fibrin unless otherwise stated. For live-cell imaging, endothelial networks were visualised over the course of 1 week using the JuLi Smart Fluorescent Cell Imager (Bulldog Bio, Portsmouth, NH, USA).

### Immunofluorescence staining

Immunofluorescence staining was performed after 1 week of co-culture if not stated otherwise. For staining of cells within a 3D fibrin-matrix, hydrogels were fixed with 4% paraformaldehyde overnight and then washed prior to adding the primary antibody diluted in 1 × phosphate-buffered saline (PBS)/1% bovine serum albumin (BSA). Primary antibody was incubated overnight and afterwards matrices were washed followed by overnight secondary antibody incubation. All steps were performed at 4 ° C on a shaker. Staining was performed against collagen type IV (rabbit-polyclonal, Abcam, Cambridge, MA, UK), laminin (rabbit-polyclonal, Abcam, Cambridge, MA, UK) or E-Selectin (BD Pharmingen, San Diego, CA, USA). Secondary antibodies goat anti-mouse Alexa Fluor 488 (Thermo Fisher Scientific Inc., Waltham, MA, USA) or goat anti-rabbit-FITC (Thermo Fisher Scientific Inc., Waltham, MA, USA) were used. For immunofluorescence staining in a two-dimensional (2D) setup, cells were fixed with 4% paraformaldehyde for 20 min. Anti-CD31-FITC (BD Pharmingen, San Diego, CA, USA) was diluted in 1 × PBS/1% BSA and incubated for 45 min. All images were taken on an epifluorescence microscope (Zeiss Observer A1, Zeiss, Oberkochen, Germany) or on a laser-scanning confocal microscope (Zeiss LSM510, Zeiss, Oberkochen, Germany).

### Measurement of fibrin degradation

Fibrin degradation was monitored using a fluorophore-conjugated fibrinogen [24]. Fibrinogen used for clot formation was spiked with Oregon green 488-labelled fibrinogen (Invitrogen, Carlsbad, CA, USA) at 5 pg per μl fibrin. To confirm the experiments using green-fluorescent fibrinogen, we additionally used Alexa Fluor 546-labelled fibrinogen (Invitrogen, Carlsbad, CA, USA) at 50 pg per μl fibrin. Concentrations were chosen after performing serial dilution measurements. With every medium change (every 2–3 days), supernatants were removed and stored at -20 °C until analyzed and fresh growth medium was added onto the fibrin matrices. To determine fluorescence, supernatants were thawed, 100 μl were transferred into opaque 96-well plates and fluorescence was recorded using a Polarstar Omega photometer (BMG Labtech, Ortenberg, Germany). The cumulative fibrin degradation after the first and after the second week was calculated and compared to aprotinin-containing scaffolds. Supernatants from four individual experiments were analysed.

### Quantification of endothelial networks

All images used for analysis were taken on an epifluorescence microscope (Zeiss Observer A1, Zeiss, Oberkochen, Germany). For network quantification, images per sample were taken and analysed as described previously [9, 15, 25]. Randomised images were processed using Adobe Photoshop CS5 (Adobe Systems, San José, CA, USA) and analysed in a blinded manner. Measurement of number of tubules and junctions was done with Angiosys software (TCS Cellworks, London, UK). Vessel diameter quantification was performed on ten vessels per image using ImageJ (NIH, Bethesda, MD, USA) [26].

### Statistics

**Fig. 1:**
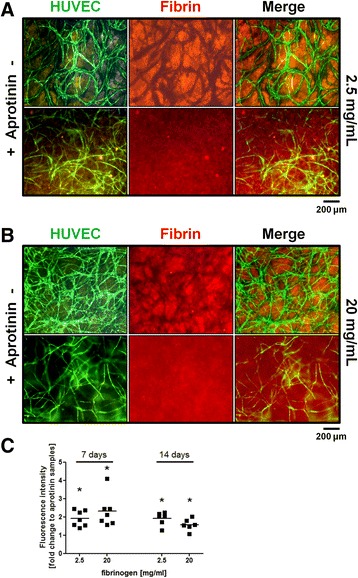
Aprotinin inhibits human umbilical vein endothelial cells (HUVEC)-induced fibrin degradation. **a, b** Images of YFP-HUVEC in co-culture with ASCs were taken after 2 weeks of incubation in clots containing 2.5 mg/mL fibrinogen (**a**) or 20 mg/mL fibrinogen (**b**) spiked with Alexa Fluor 546-labelled fibrinogen. Locations with high fibrinolytic activity can be seen as dark tube-like structures in fibrin in aprotinin-free conditions. **c** Supernatants were collected from HUVEC and ASC embedded in fluorescent fibrin clots, measured and the accumulated fluorescence of 7 days of culture was calculated. Supernatant from clots cultured in aprotinin-free medium showed a higher fluorescence signal compared to clots incubated in aprotinin-containing medium. Values represent four individual experiments and fold changes of aprotinin-free samples compared to aprotinin-containing samples. *n* = 7 (7 days); *n* = 6 (14 days); *p < 0.05. Scale bar: 200 μm

**Fig. 2:**
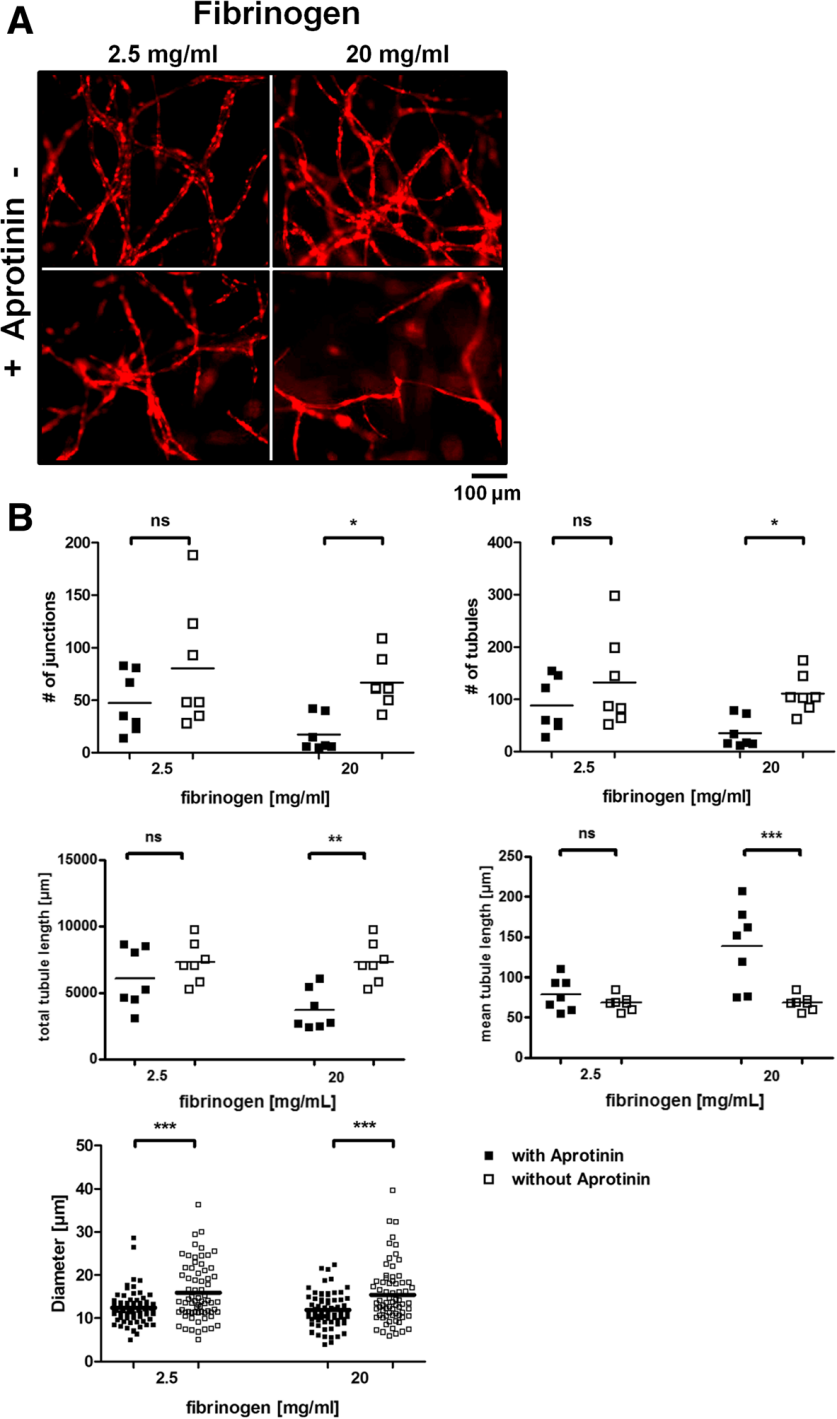
Addition of aprotinin to co-culture fibrin matrices influences vascular network density and vessel diameter. **a** Images of mCherry-HUVEC were taken after 2 weeks of incubation in clots containing either 2.5 mg/mL or 20 mg/mL fibrinogen. **b** Quantification of vascular networks by determining the number of tubules, junctions, total and mean tubule length and the vessel diameter. HUVEC cultured in aprotinin-free medium showed a higher number of tubules, junctions and total tubule length and a lower mean tubule length, which was significant in clots containing 20 mg/mL fibrinogen. Additionally, vessel diameter was significantly increased in aprotinin-free conditions. Values are from two independent experiments; *n* = 7 (junctions and tubules); *n* = 70 (diameter); *p < 0.05; ***p < 0.001; ns = not significant. Scale bar: 100 μm

**Fig. 3:**
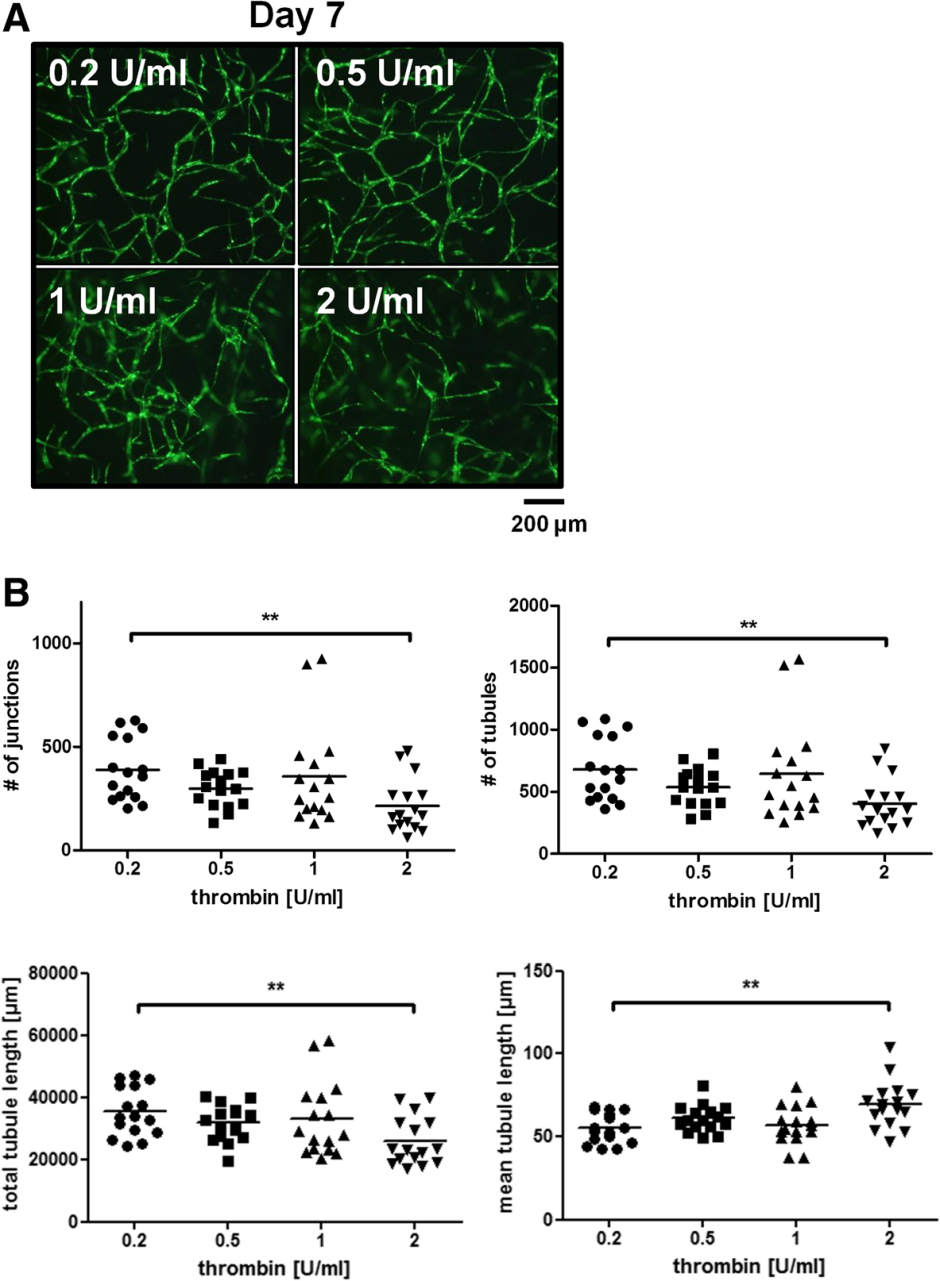
The influence of thrombin on vascular structures. **a** Vascular network formation could be observed in any sample independent of the thrombin concentration used. **b** Significant differences in number of vascular network parameters could only be detected if 2 U/ml thrombin was used. In these matrices, number of junctions and tubules and total tubule length were significantly lower and mean tubule length was significantly higher compared to samples prepared with 0.2 U/ml thrombin. No aprotinin was used in any sample. *n* = 8 from two independent experiments; **p < 0.01. Scale bar: 200 μm

**Fig. 4:**
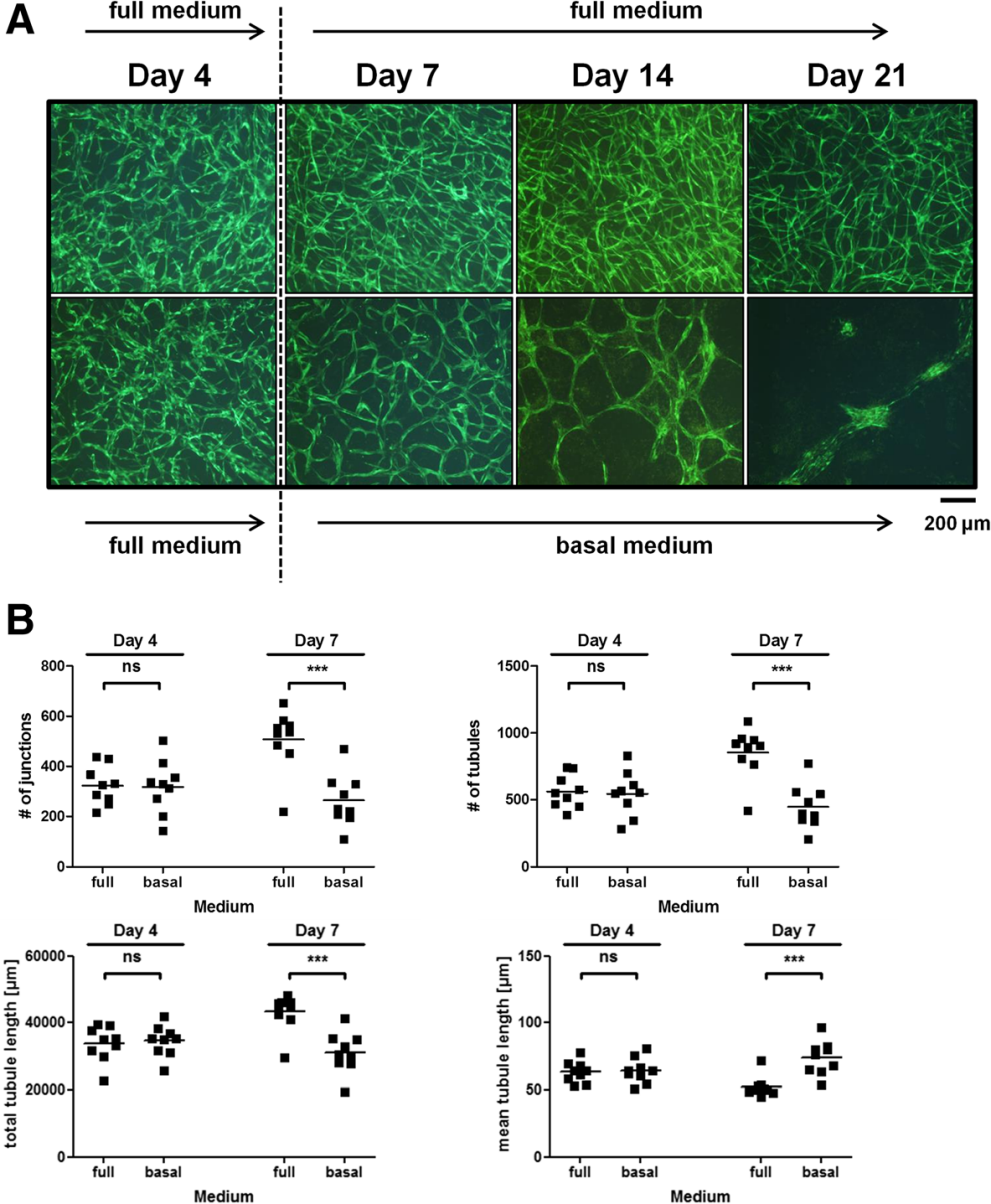
Effect of medium supplements on established vascular networks. Fibrin matrices containing HUVEC and ASC were cultured for 4 days in full medium. Afterwards, co-culture in fibrin scaffolds was either continued in full medium or switched to basal medium. No aprotinin was added. **a** Cells in fibrin clots cultured exclusively in full medium formed vascular networks within 4 days and the vascular structures persist over 21 days. In samples that were switched to basal medium, degradation of the fibrin matrix could be observed on day 14. No difference in vascular network density was visible on day 4 before the switch occurred. **b** Quantification of network formation. Co-cultures displayed similar vascular network values until the switch from full to basal medium. Co-culture in basal medium results in significantly less junctions, tubules, decreased total tubule length and increased mean tubule length compared to full medium samples on day 7. Values are from two independent experiments using two different ASC donors; *n* = 9; ***p < 0.001; *ns* not significant. Scale bar: 200 μm

**Fig. 5:**
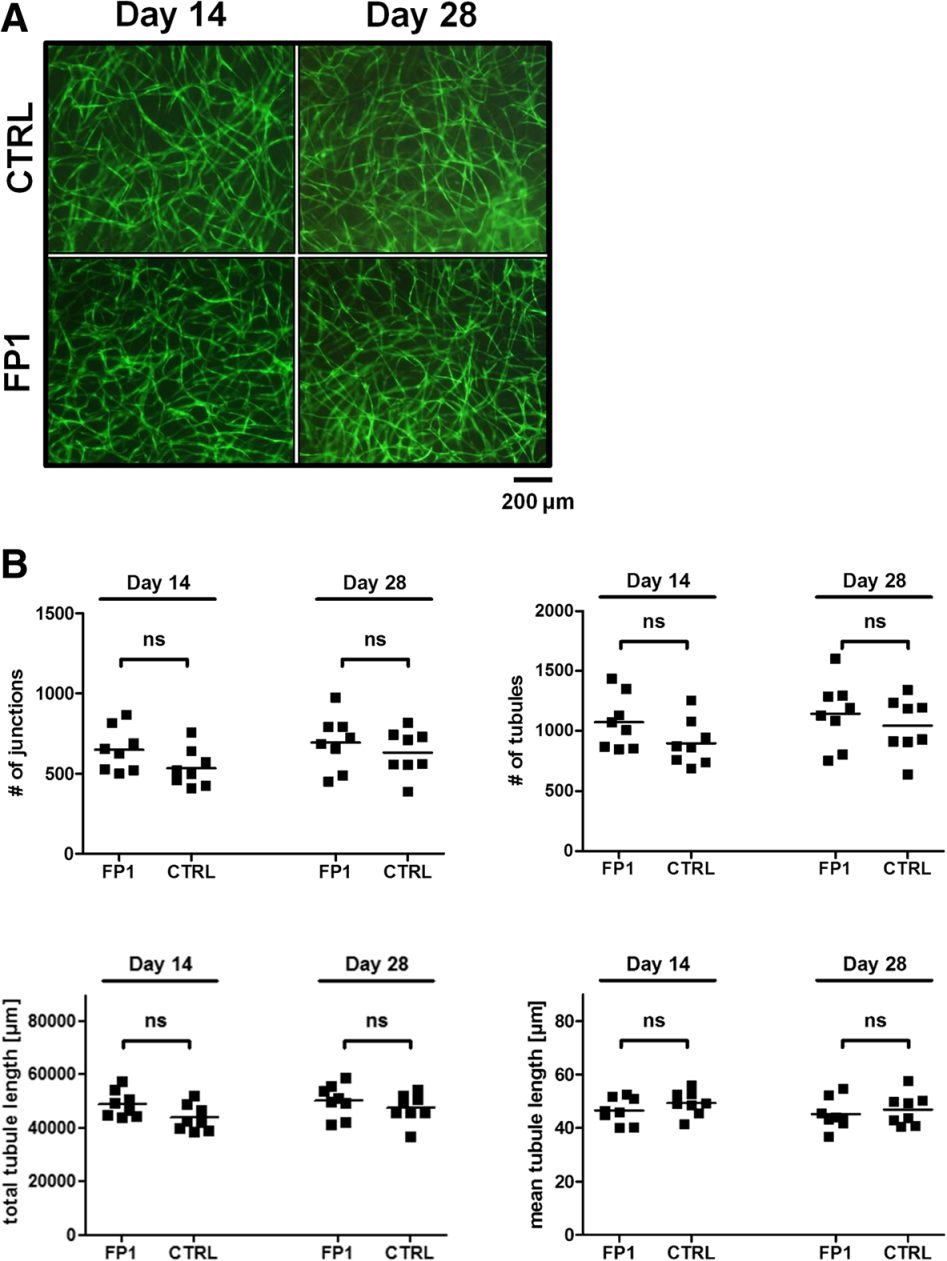
The influence of different fibrinogen formulations on vascular structures. **a** When comparing our standard fibrinogen (CTRL) versus another fibrinogen formulation (FP1), we did not observe an effect on vascular network formation. **b** No significant difference in number of vascular network parameters could be observed in any sample. All samples were cultured without aprotinin. n = 8 from one experiment; *ns* not significant. Scale bar: 200 μm

Statistical evaluations were performed with GraphPad Prism 5 software (GraphPad Software, San Diego, CA, USA). Fold changes in Fig. 1 were analysed by two-tailed Wilcoxon matched-pair signed-rank test. Data shown in Figs. 2, 4 and 5 were analysed by two-way analysis of variance (ANOVA). Significance of data in Fig. 3 was determined using one-way ANOVA. Values were considered significant when *p* < 0.05.

## Results

### Aprotinin in cell culture supernatant inhibits fibrin degradation

To investigate the influence of aprotinin on fibrinolysis, we visualised and quantified fibrin degradation by employing fluorophore-labelled fibrinogen, since measured fluorescence in the supernatant correlates with fibrin degradation [24]. Sites with a high fibrinolytic activity could be visualised as locations with low fluorescence signal in scaffolds containing either 2.5 mg/ml (Fig. 1a) or 20 mg/ml fibrinogen (Fig. 1b). These sites co-localise with vascular structures formed by HUVEC in co-culture with ASCs. A uniform fluorescence could be seen in all samples containing aprotinin, indicating that fibrin was barely degraded around vascular tubules. We observed a significant increase in fold change fluorescence in supernatants from samples that did not contain aprotinin compared to aprotinin-containing samples (Fig. 1c). Specifically, in aprotinin-free supernatants from matrices containing 2.5 mg/ml fibrinogen, we observed on average a 1.9-fold increase in fluorescence after both the first week and the second week of incubation compared to aprotinin-containing samples. When cells were cultured in matrices containing 20 mg/ml fibrinogen, the fluorescence intensity of supernatants from these samples increased on average by 2.3-fold after the first 7 days and by 1.5-fold after the second 7 days of culture compared to aprotinin-containing samples.

### Inhibition of fibrinolysis impairs vascular network formation

To determine if the observed inhibition of fibrin degradation has an influence on vascular network formation, we performed co-culture experiments to quantify the number of junctions, tubules and the vessel diameter. Aprotinin-free co-culture of HUVEC and ASC embedded in 2.5 mg/ml fibrin scaffolds led to an increased vessel density (Fig. 2a). This effect was even more pronounced in scaffolds containing 20 mg/ml fibrinogen. Quantification of vascular networks revealed an increase in number of junctions and tubules in 2.5 mg/ml fibrinogen scaffolds (47.43 vs. 80.43 mean number of junctions and 88.14 vs. 132.6 mean number of tubules), which was significant when scaffolds contained 20 mg/ml fibrinogen compared to respective samples without aprotinin (17.29 vs. 66.86 mean number of junctions and 35.14 vs. 111.0 mean number of tubules). Accordingly, total tubule length was significantly increased in aprotinin-free 20 mg/ml fibrin clots compared to aprotinin-containing clots while mean tubule length was significantly decreased indicating that more branches have formed in these samples. No difference in total tubule length and mean tubule length was observed in samples with 2.5 mg/ml fibrinogen between aprotinin-free and aprotinin-containing samples. We furthermore found that tube-like structures were significantly thicker (12.39 vs. 15.88 μm in 2.5 mg/ml and 11.89 vs. 15.40 μm average thickness in 20 mg/ml fibrinogen scaffolds) in aprotinin-free conditions independent of the fibrinogen concentration used (Fig. 2b). However, despite the effects of aprotinin on vascular network formation, we could show that HUVEC actively form vascular networks as evidenced by live-cell imaging over the course of the first week of incubation (see Additional file 1). Additionally, long-term co-culture of HUVEC with ASC is possible in fibrin clots cultured with 100 KIU/ml aprotinin and containing 2.5 mg/ml or 20 mg/ml fibrinogen. Vascular structures are still visible after 15 weeks of culture (see Additional file 2).

### Aprotinin influences vascular network formation in a dose-dependent manner

We further investigated the dose-dependency of aprotinin by preparing fibrin scaffolds containing ASC and HUVEC and culturing them in different aprotinin concentrations for 28 days as shown in Additional file 3. Samples without aprotinin had the highest vascular network density shown by number of junctions, tubules and total tubule length. The mean tubule length increased dose-dependently reaching a peak at 20 KIU/ml aprotinin. A concentration of 5 KIU/ml already caused a visible change in vascular network parameters. There was no difference in number of junctions and tubules when using concentrations reaching from 10 to 100 KIU/ml.

### Aprotinin does not influence network formation in a fibrin-free environment

To investigate the influence of fibrin on endothelial network formation, HUVEC were co-cultured with ASC for 1 week without a fibrin matrix. Cells were then fixed and stained against CD31 to reveal structures formed by HUVEC. ECFC were used to confirm HUVEC results. These results can be seen in Additional file 4. Both HUVEC and ECFC showed network formation in co-culture with ASC but without fibrin. We additionally used this fibrin-free 2D co-culture set-up to exclude a direct effect of aprotinin on network morphology. Staining against CD31 after 1 week of co-culture showed that HUVEC formed vessel-like structures, independent of the presence of aprotinin (100 KIU/ml) in the medium. This indicates that aprotinin specifically influences vascular network formation by interfering with fibrin degradation.

### Thrombin concentrations influence network formation

We have previously reported the influence of different fibrinogen concentrations on vascular network formation [15]. Since fibrin polymerisation depends on thrombin, we determined the effects of different thrombin concentrations (0.2, 0.5, 1 and 2 U/ml) on network formation. We found that concentrations up to 1 U/ml did not influence network formation while the use of 2 U/ml thrombin decreased the observed vessel density (Fig. 3a). Number of junctions, tubules, total as well as mean tubule length showed no significant differences except at a thrombin concentration of 2 U/ml. Fibrin matrices for which 2 U/ml thrombin was used showed a significantly lower number of junctions and tubules and total tubule length and a significantly higher mean tubule length compared to matrices prepared with 0.2 U/ml (Fig. 3b). Since thrombin is known to activate EC by protease-activated receptors (PARs) [27], HUVEC were embedded into fibrin scaffolds together with ASC and stained for the inflammatory surface marker E-selectin at four different time points (4 h, 1 day, 4 and 7 days). The thrombin concentration used was 0.2 U/ml. Fibrin scaffolds were additionally treated with 10 ng/ml tumour necrosis factor alpha (TNF-α) as positive control. Staining against E-selectin revealed transient activation of HUVEC after 4 h while no positive staining could be seen after 1 day of incubation. TNF-α led to a strong increase of E-selectin expression after 4 h which declined after 1 day. No positive E-selectin staining could be observed after 4 or 7 days in any sample (see Additional file 5).

### Basal medium conditions do not support vascular network formation

We have recently shown that vascular networks can be maintained in basal medium for up to 14 days [15]. Here, we repeated the experiment without aprotinin to determine its influence under basal culture conditions. Fibrin clots were first cultured in full medium containing serum and growth factors for up to 4 days and then switched to basal medium for additional 17 days. Cultivation in basal medium ultimately led to a visible decrease in number of junctions and tubules starting at day 7. Continuous incubation in full medium, on the other hand, resulted in formation and maintenance of dense vessel-like structures. Specifically, fibrin matrix degradation was observable in basal medium conditions after 14 days while the fibrin scaffold remained intact in full medium culture conditions (Fig. 4a). Quantification of vascular network parameters shows the similarity of all samples on day 4 of incubation where the medium switch occurred. However, after 3 additional days of incubation, we observed that samples cultured in full medium had a significantly higher number of junctions and tubules compared to samples cultured in basal medium. Furthermore, total tubule length was significantly increased while mean tubule length was significantly decreased in full medium-cultured samples compared to basal medium-cultured clots (Fig. 4b). No later time points were included in the quantification since the degraded matrix would be a confounding factor for image analysis.

### The influence of fibrinogen formulation on vascular structures

To exclude an effect of additional fibrin scaffold components such as fibronectin, factor XIII and plasminogen we tested a different fibrinogen formulation (FP1) in which fibrinogen is the only protein component as stated in the Methods section. No significant difference in vascular structure morphology (Fig. 5a) as well as number of junctions, number of tubules, total or mean tubule length could be observed in vascular structures formed in fibrin clots that were prepared with different fibrinogen products (Fig. 5b).

### EC-ASC co-cultures in fibrin deposit collagen type IV and laminin independent of aprotinin addition

**Fig. 6:**
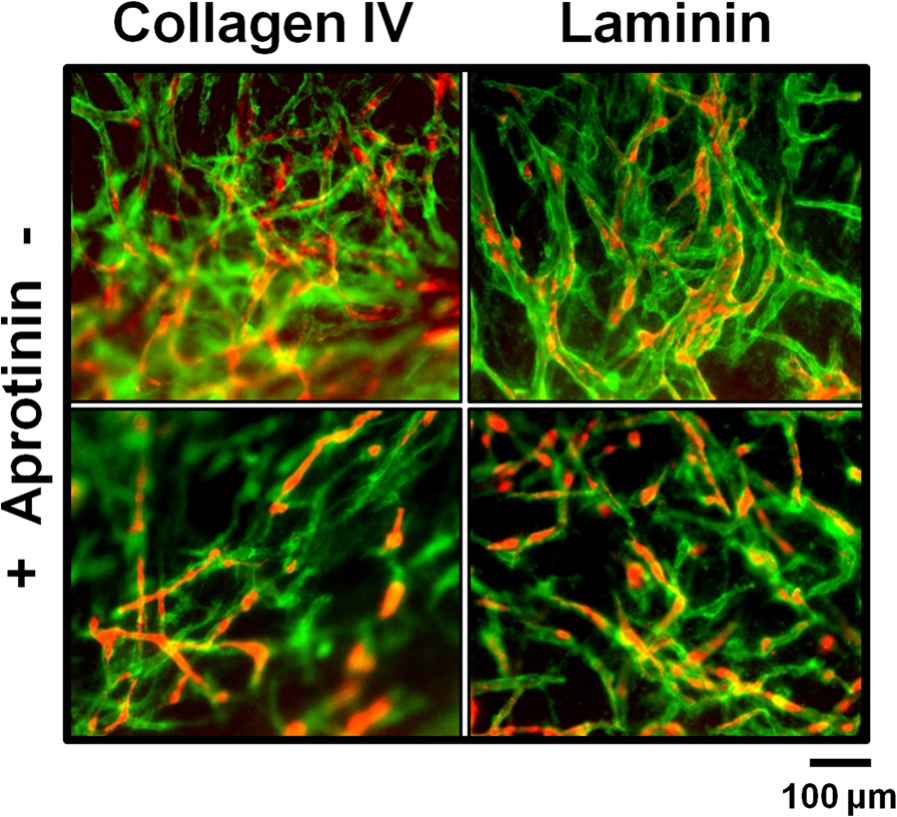
Production of collagen type IV and laminin by HUVEC under the influence of aprotinin. mCherry-HUVEC are depicted in *red*, collagen type IV and laminin in *green*. mCherry-HUVEC were co-cultured with ASC in a fibrin matrix. Collagen type IV as well as laminin is clearly visible in close proximity to tube-like structures of mCherry-HUVEC in matrices with as well as without aprotinin. Scale bar: 100 μm

Physiological microvasculature is surrounded by several extracellular matrix (ECM) components, with collagen type IV and laminin being the most prominent proteins. We performed immunofluorescence staining against collagen type IV as well as laminin in order to check for ECM formation and to examine if ECM production is aprotinin-dependent. mCherry-HUVEC (red) that were co-cultured with ASC in fibrin clots formed vascular-networks after 1 week of incubation. Staining against collagen type IV (green) as well as laminin (green) revealed their location to be in close proximity around vessel-like structures (Fig. 6). The addition of aprotinin (100 KIU/ml) showed no qualitative effect on ECM production.

## Discussion

In this study we show the influence of aprotinin and thrombin on vascular networks derived from co-cultures of HUVEC and ASC in a fibrin matrix. Furthermore, our results here and previously published data by our group imply that aprotinin, while impairing vascular network density, may be necessary for culturing endothelial capillary networks in serum- and growth factor-free medium [15]. In general, thorough characterisation of these influences on co-cultures is of crucial importance for applications towards both microvascular tissue engineering and drug testing in preclinical research. In either case, engineered vasculature has to reflect the normal physiology in vivo and thus functionality in terms of providing adequate blood flow. As “gold standard”, the integration of HUVEC with supporting cell types in a fibrin matrix has been the focus of many studies mainly by the Tranquillo, Putnam and Levenberg groups [13, 28, 29]. Microvascular endothelial cells from human dermis and ECFCs represent other endothelial cell types frequently used for in vitro vascularisation experiments in fibrin [12, 13]. We initially used ECFC and ASC in our co-culture system to show the potential use of this generated microvasculature in an autologous setting [7, 9]. Both cell types can be easily harvested from one and the same individual using peripheral blood and fat tissue. Furthermore, we recently demonstrated the in vivo functionality of ECFC/ASC-co-cultures in fibrin by subcutaneous implantation in nude mice and perfusion with a tail vein-injected fluorescent dye [4]. Other groups as well showed the functionality and anastomosis of vascular structures derived from co-cultures in vivo (prevascularised HUVEC and ASC spheroids [30] or collagen matrices containing endothelial progenitor cells (EPC) and ASC [31]). However, discrepancies exist between HUVEC and ECFC in terms of gene expression as determined by gene array analysis [32]. Nevertheless, we showed previously that vascular network formation occurs when either cell type is co-cultured with ASC in fibrin [9], suggesting that HUVEC can be used as a surrogate for ECFC in our vascularisation model. Additionally, we show here that both HUVEC and ECFC form tube-like structures in a 2D co-culture as evidenced by CD31 staining.

Besides its role as sealant in clinical practice, fibrin as a scaffold and biomaterial has been extensively used for diverse tissue engineering applications [33]. For fibrin polymerisation addition of thrombin is necessary. Importantly, thrombin is known to activate endothelial cells and elicit inflammatory responses [27]. To exclude long-term negative effects on the microvascular structures we performed an immunofluorescence staining using anti-E-selectin antibody and show a slight upregulation of this marker shortly after embedding the cells in the matrix. However, when cells start to form vascular tubes, E-selectin expression was not detectable anymore, thus excluding long-term inflammatory activation of fibrin-embedded EC by thrombin. Additionally, its biological features make fibrin ideally suitable for soft tissue engineering approaches, especially considering the integration of microvasculature in engineered tissues [12]. Our results demonstrate that a vascular network can be established in vitro and that fibrinolysis occurs where vessel-like structures are found. Degradation of fibrin can be mediated by the proteolytic activity of the serine protease plasmin [34]. When stimulated by angiogenic factors such as vascular endothelial growth factor (VEGF), endothelial cells can initiate the activation of the plasmin precursor plasminogen by releasing tissue-type plasminogen activator or urokinase plasminogen activator (tPA or uPA), ultimately leading to plasmin generation and activation, and fibrinolysis [35, 36]. We and others have previously shown that ASC secrete high levels of VEGF [9, 37], suggesting that this effect potentially causes the co-localisation of vascular structures with sites of high fibrinolytic activity, as endothelial cells initiate the activation of plasminogen.

However, methods to prolong fibrin stability are required for certain approaches such as therapeutic growth factor delivery since growth factors are released in lower doses and over longer time periods [38]. One possibility to increase the stability of fibrin scaffolds is the addition of anti-fibrinolytic agents. Our results and previously published reports show that aprotinin, a serine protease inhibitor, can significantly reduce fibrin degradation both in vitro and in vivo [17, 18, 39]. Although widely used, the addition of aprotinin as an anti-fibrinolytic agent remains controversial. When administered systemically in patients undergoing cardiac surgery, the use of aprotinin results in a higher mortality rate when compared to other anti-fibrinolytic agents [40, 41]. As a result, the US Food and Drug Administration agreed with the manufacturer to remove all remaining stocks of Trasylol® (aprotinin injection) [42]. This may be the reason why the compatibility of alternative agents to inhibit fibrin degradation was tested in in vitro experiments, despite that different concentrations were used in clinics [16]. However, since our results indicate that specifically inhibition of fibrinolysis is responsible for the reduction in vessel density, the use of other agents that inhibit plasmin-mediated fibrinolysis might lead to a similar impairment of vascularisation of fibrin scaffolds. Data to support this statement were published in another study where it was shown that VEGF-induced capillary formation in fibrin can be impaired by either the addition of aprotinin or by the removal of plasminogen from the fibrin matrix [20]. It could therefore be hypothesised that plasminogen-driven fibrinolysis is key to vascularisation of fibrin scaffolds.

Alternatively, fibrin can be degraded by matrix metalloproteinase (MMP) activity, indicating that vascularisation of fibrin scaffolds does not depend on plasminogen [43, 44]. Notably, it has been shown that endothelial cells from both plasminogen-deficient and wild-type mice were capable of neovascularising plasminogen-free fibrin both in vitro and in vivo [45]. These reports conclude that endothelial MMP activity and not plasminogen-driven fibrinolysis are key to vascularisation of fibrin hydrogels. This would certainly enable the use of aprotinin to enhance scaffold stiffness as we and others have previously shown that endothelial cells sprouting into the scaffold express MMP14 to degrade fibrin despite being treated with aprotinin suggesting that aprotinin has no effect on MMP activity [7, 43]. However, the key difference to our data shown here is that the experiments performed by other groups were done using exclusively plasminogen-depleted but factor XIII (FXIII)-containing fibrin [43–46]. We, on the other hand, have tested here the effect of plasminogen-free compared to plasminogen-containing fibrin scaffolds on vascular network formation, yet, without finding significant differences. It should be noted that the fibrinogen formulation tested here is not only free of plasminogen but also free of FXIII which crosslinks fibrin fibres. Addition of FXIII to fibrin clots in vitro results in a significantly higher fibrin clot stiffness compared to unmodified samples after cell-free incubation for 10 days, despite the initial clot stiffness being similar in all samples [19]. This observation suggests that fibrin degradation was slowed down. Therefore, while our tested fibrinogen formulation does not contain plasminogen supposedly resulting in impaired fibrinolysis, it does also not contain FXIII, which in turn may in part compensate for the absence of plasminogen. Indeed, it has been shown that FXIII impairs sprouting of endothelial cells embedded in fibrin in a dose-dependent manner [47]. Hence, the vessel density is not significantly different when compared to our control fibrinogen formulation. To verify this hypothesis, additional experiments with fibrinogen scaffolds containing plasminogen but not FXIII would need to be performed. In addition, we could show that a dense vascular network can form in high fibrinogen and thrombin concentrations in absence of aprotinin. Furthermore, high fibrinogen concentrations results in increased fibrin gel stiffness. It has been shown that the structural modulus of fibrin gels containing 2 mg/ml fibrinogen is significantly lower compared to gels containing 17 mg/ml fibrinogen while maintaining the same thrombin concentration [11]. Our results suggest that vascular network density does not need to come at the expense of scaffold stiffness, which is crucial for in vivo applications [4]. The inhibition of fibrinolysis by aprotinin can be necessary in some tissue engineering applications. Whenever scaffold stability in vivo is required, fibrinolysis by invading cells needs to be reduced [17, 18]. As mentioned before, therapeutic growth factor delivery requires long-lasting matrices that withstand degradation [48]. Scaffold degradation and tissue regrowth are interdependent processes [49], despite recent studies describing matrix degradation and loss of volume as negative and unwanted features in in vivo situations [18, 19]. While it has been reported that subcutaneous implantation of scaffolds without aprotinin results in volume loss and lower number of invading blood vessels [18], skin tissue could have regenerated faster since scaffold degradation was not impaired. The parameter of tissue regeneration, however, has not been investigated in this study and would therefore need to be further analysed. Consequently, another report demonstrates that the use of aprotinin in vivo results in delayed wound healing as granulation tissue did not fully regenerate [50]. Besides its inhibitory effect on fibrinolysis, it cannot be ruled out that aprotinin has additional effects on cell physiology. However, when we co-cultured endothelial cells with ASC in a 2D setting without fibrin we did not find any influence of aprotinin on tube-like structure formation, suggesting that the described effects in our 3D setting are primarily related to fibrinolysis inhibition. Furthermore, we show that ECM deposition indicated by collagen type IV and laminin staining is unaffected by aprotinin in fibrin clots. Interestingly, it has been reported that aprotinin can even enhance angiogenesis in absence of fibrin as demonstrated using a chorioallantoic membrane (CAM) assay [51]. These results suggest that the reduction of vascular network density in 3D fibrin scaffolds by aprotinin is specifically due to fibrinolysis inhibition. Classically, endothelial cells are cultured in cell culture medium containing several growth factors and a varying amount of fetal calf serum. We have previously demonstrated the suitability of human-derived platelet lysate as a substitute for fetal calf serum in endothelial cell culture suggesting that certain medium components are exchangeable without affecting vascularisation efficacy [8]. Here we analysed the formation of vascular structures in full medium containing serum and several growth factors *versus* basal medium. We found most junctions in the vascular network in the full medium condition, while network density decreased to a level where it could not be quantified anymore in samples cultured without serum and growth factors. This decrease in vessel density seems to be specifically related to scaffold degradation as we have previously demonstrated that the vascular network remains stable in basal medium when aprotinin is added [15]. ASC are the primary source of growth factors necessary for vessel-like structure formation in fibrin [9]. This cell type has been shown to promote endothelial-mediated fibrinolysis via the plasmin pathway in contrast to bone marrow-derived stem cells which stimulate MMP-mediated ECM degradation [52, 53]. ASC can furthermore regulate endothelial cell-mediated fibrin degradation by secretion of several tissue inhibitors of metalloproteinases (TIMP) and plasminogen activator inhibitor-1 (PAI-1) [9, 54, 55]. Influences on the ASC secretome would therefore inevitably affect stability of fibrin scaffolds and therefore vascularisation. Indeed, it has been shown that growth factor and serum-free medium results in significant changes on ASC proliferation, gene expression and growth factor secretion [56, 57]. However, it remains unknown which proteins are influenced by this change in our co-culture model. Still, factors specifically involved in fibrin degradation seem to be at least in part influenced by co-culture in this medium formulation.

In this study. we used mainly HUVEC as they represent a “gold standard” in the field of angiogenesis research. However, these cells are not clinically applicable and thus not suitable for regenerative medicine purposes. We found vascular network formation also with ECFC derived from iPS cells. In addition, we have previously shown network formation of ECFC from peripheral blood and ASC in a fibrin matrix, both cell types can be used in an autologous setting [7]. Moreover, vascular network formation in fibrin has been also demonstrated using microvascular endothelial cells from dermis and adipose tissue [58], thus suggesting vascular network formation in fibrin as a stable phenomenon, which might be critical for organotypic vessels and thus have general implications for vascular regenerative medicine.

## Conclusions

Our data demonstrate that aprotinin-mediated stabilisation of fibrin leads to impaired vascularisation presumably by inhibition of plasminogen activation rather than generally affecting network formation or ECM deposition. This indicates that fibrinolysis inhibition by aprotinin is a crucial parameter for vascularisation strategies using co-culture models. Based on our data, we furthermore propose that fibrinolysis inhibition is required for maintaining vascularised fibrin scaffolds in serum- and growth factor-free conditions, thereby fulfilling an important prerequisite for clinical translation of prevascularised tissues. Taken together, our findings contribute to the understanding how modulation of fibrinolysis affects in vitro models for vascularisation of engineered constructs in fibrin.

## Additional files

Additional file 1: GFP-HUVEC actively form a vascular network in co-culture with ASC in the presence of aprotinin. Live-cell imaging of GFP-HUVEC embedded in fibrin with ASC reveals vascular network formation over the course of 1 week. (AVI 17466 kb)

Additional file 2: Co-cultures of HUVEC and ASC can be maintained in culture up to 15 weeks. (A) HUVEC and ASC were embedded in fibrin matrices containing 2.5 mg/mL fibrinogen. (B) Co-culture fibrin matrices containing 20 mg/mL were maintained in culture for up to 15 weeks. All images are representative for fibrin matrices from three independent experiments. Aprotinin was used in all samples. Scale bar: 200 μm. (DOC 1344 kb)

Additional file 3: Influence of different aprotinin concentrations on HUVEC network formation. (A) Representative images of the effect of different aprotinin concentrations (0 KIU/ml, 5 KIU/ml, 10 KIU/ml, 20 KIU/ml, 30 KIU/ml and 100 KIU/ml) on HUVEC/ASC vascular network formation taken on day 28 of incubation. (B) Quantification of the network by number of junctions, tubules, total and mean tubule length. Increased aprotinin concentration results in a decreased number of tubules as well as junctions and total tubule length. Mean tubule length shows a dose-dependent increase, which peaks in samples with 20 KIU/ml aprotinin. Values are from two independent experiments using two different ASC donors; *n* = 2. Scale bar: 200 μm. (DOC 741 kb)

Additional file 4: Aprotinin does not impair network formation of endothelial cells in a 2D setup. CD31 staining of endothelial cells (either HUVEC or ECFC) reveals tube-like structures when co-cultured with ASC in a 2D-setup devoid of fibrin. Aprotinin (100 KIU/ml) has no effect on HUVEC 2D tube formation. Scale bar: 100 μm or 50 μm as indicated. (DOC 370 kb)

Additional file 5: Thrombin transiently activates EC. HUVEC-ASC clots were fixed and immunofluorescence was performed against E-Selectin after 4 h, 1 day, 4 days and 7 days. Staining revealed transient activation of EC via thrombin after 4 h. TNF-α (10 ng/ml), serving as a positive control, activated EC more strongly after 4 h than thrombin alone. No aprotinin was used in any sample. *n* = 8 from two independent experiments. Scale bar: 200 μm. (DOC 322 kb)

## Abbreviations

2D: Two-dimensional
3D: Three-dimensional
ANOVA: Analysis of variance
ASC: Adipose-derived stem cells
BSA: Bovine serum albumin
CAM: Chorioallantoic membrane
EBM-2: Endothelial basal medium-2
EC: Endothelial cells
ECFC: Endothelial colony-forming cells
ECM: Extracellular matrix
EGF: Epidermal growth factor
EGM-2: Endothelial growth medium-2
FCS: Fetal calf serum
FGF: Fibroblast growth factor
FXIII: Factor XIII
GA-1000: Gentamicin/amphotericin-B
HUVEC: Human umbilical vein endothelial cells
LSM 1077: Lymphocyte separation medium
MMP: Matrix metalloproteinase
PAI-1: Plasminogen activator inhibitor-1
PAR: Protease-activated receptor
PBS: Phosphate-buffered saline
R3-IGF-1: R3-insulin-like growth factor-1
TIMP: Tissue inhibitor of metalloproteinase
TNF-α: Tumour necrosis factor alpha
t-PA: Tissue plasminogen activator
u-PA: Urokinase plasminogen activator
VEGF: Vascular endothelial growth factor

## References

[1] Mao AS, Mooney DJ (2015). Regenerative medicine: current therapies and future directions. Proc Natl Acad Sci.

[2] Muehleder S, Ovsianikov A, Zipperle J, Redl H, Holnthoner W (2014). Connections matter: channeled hydrogels to improve vascularization. Front Bioeng Biotechnol.

[3] Schneider KH, Aigner P, Holnthoner W, Monforte X, Nürnberger S, Rünzler D (2016). Decellularized human placenta chorion matrix as a favorable source of small-diameter vascular grafts. Acta Biomater.

[4] Pill K, Hofmann S, Redl H, Holnthoner W (2015). Vascularization mediated by mesenchymal stem cells from bone marrow and adipose tissue: a comparison. Cell Regen.

[5] Newman AC, Nakatsu MN, Chou W, Gershon PD, Hughes CCW (2011). The requirement for fibroblasts in angiogenesis: fibroblast-derived matrix proteins are essential for endothelial cell lumen formation. Mol Biol Cell.

[6] Ghanaati S, Unger RE, Webber MJ, Barbeck M, Orth C, Kirkpatrick JA (2011). Scaffold vascularization in vivo driven by primary human osteoblasts in concert with host inflammatory cells. Biomaterials.

[7] Holnthoner W, Hohenegger K, Husa A-M, Muehleder S, Meinl A, Peterbauer-Scherb A (2015). Adipose-derived stem cells induce vascular tube formation of outgrowth endothelial cells in a fibrin matrix. J Tissue Eng Regen Med.

[8] Hofbauer P, Riedl S, Witzeneder K, Hildner F, Wolbank S, Groeger M (2014). Human platelet lysate is a feasible candidate to replace fetal calf serum as medium supplement for blood vascular and lymphatic endothelial cells. Cytotherapy.

[9] Rohringer S, Hofbauer P, Schneider KH, Husa A-M, Feichtinger G, Peterbauer-Scherb A (2014). Mechanisms of vasculogenesis in 3D fibrin matrices mediated by the interaction of adipose-derived stem cells and endothelial cells. Angiogenesis.

[10] Chan BP, Leong KW (2008). Scaffolding in tissue engineering: General approaches and tissue-specific considerations. Eur Spine J.

[11] Duong H, Wu B, Tawil B (2009). Modulation of 3D fibrin matrix stiffness by intrinsic fibrinogen-thrombin compositions and by extrinsic cellular activity. Tissue Eng Part A.

[12] van Hinsbergh VW, Collen A, Koolwijk P (2001). Role of fibrin matrix in angiogenesis. Ann N Y Acad Sci.

[13] Morin KT, Tranquillo RT (2013). In vitro models of angiogenesis and vasculogenesis in fibrin gel. Exp Cell Res.

[14] Unger C, Skottman H, Blomberg P, Dilber MS, Hovatta O (2008). Good manufacturing practice and clinical-grade human embryonic stem cell lines. Hum Mol Genet.

[15] Hasenberg T, Mühleder S, Dotzler A, Bauer S, Labuda K, Holnthoner W (2015). Emulating human microcapillaries in a multi-organ-chip platform. J Biotechnol.

[16] Cholewinski E, Dietrich M, Flanagan TC, Schmitz-Rode T, Jockenhoevel S (2009). Tranexamic acid--an alternative to aprotinin in fibrin-based cardiovascular tissue engineering. Tissue Eng Part A.

[17] Lorentz KM, Kontos S, Frey P, Hubbell JA (2011). Engineered aprotinin for improved stability of fibrin biomaterials. Biomaterials.

[18] Thomson KS, Dupras SK, Murry CE, Scatena M, Regnier M (2014). Proangiogenic microtemplated fibrin scaffolds containing aprotinin promote improved wound healing responses. Angiogenesis.

[19] Thomson KS, Korte FS, Giachelli CM, Ratner BD, Regnier M, Scatena M (2013). Prevascularized microtemplated fibrin scaffolds for cardiac tissue engineering applications. Tissue Eng Part A.

[20] Collen A, Hanemaaijer R, Lupu F, Quax PHA, Van Lent N, Grimbergen J (2003). Membrane-type matrix metalloproteinase-mediated angiogenesis in a fibrin-collagen matrix. Blood.

[21] Lesman A, Koffler J, Atlas R, Blinder YJ, Kam Z, Levenberg S (2011). Engineering vessel-like networks within multicellular fibrin-based constructs. Biomaterials.

[22] Jockenhoevel S, Zund G, Hoerstrup SP, Chalabi K, Sachweh JS, Demircan L (2001). Fibrin gel - advantages of a new scaffold in cardiovascular tissue engineering. Eur J Cardio-thoracic Surg.

[23] Wolbank S, Peterbauer A, Fahrner M, Hennerbichler S, van Griensven M, Stadler G (2007). Dose-dependent immunomodulatory effect of human stem cells from amniotic membrane: a comparison with human mesenchymal stem cells from adipose tissue. Tissue Eng.

[24] Wolbank S, Pichler V, Ferguson JC, Meinl A, van Griensven M, Goppelt A (2015). Non-invasive in vivo tracking of fibrin degradation by fluorescence imaging. J Tissue Eng Regen Med.

[25] Charwat V, Schütze K, Holnthoner W, Lavrentieva A, Gangnus R, Hofbauer P (2015). Potential and limitations of microscopy and Raman spectroscopy for live-cell analysis of 3D cell cultures. J Biotechnol.

[26] Schneider CA, Rasband WS, Eliceiri KW (2012). NIH Image to ImageJ: 25 years of image analysis. Nat Methods.

[27] Kaplanski G, Marin V, Fabrigoule M, Boulay V, Benoliel AM, Bongrand P (1998). Thrombin-activated human endothelial cells support monocyte adhesion in vitro following expression of intercellular adhesion molecule-1 (ICAM- 1; CD54) and vascular cell adhesion molecule-1 (VCAM-1; CD106). Blood.

[28] Carrion B, Kong YP, Kaigler D, Putnam AJ (2013). Bone marrow-derived mesenchymal stem cells enhance angiogenesis via their α6β1 integrin receptor. Exp Cell Res.

[29] Blinder YJ, Freiman A, Raindel N, Mooney DJ, Levenberg S (2015). Vasculogenic dynamics in 3D engineered tissue constructs. Sci Rep.

[30] Verseijden F, Posthumus-van Sluijs SJ, Farrell E, van Neck JW, Hovius SER, Hofer SOP (2010). Prevascular structures promote vascularization in engineered human adipose tissue constructs upon implantation. Cell Transplant.

[31] Traktuev DO, Prater DN, Merfeld-Clauss S, Sanjeevaiah AR, Saadatzadeh MR, Murphy M (2009). Robust functional vascular network formation in vivo by cooperation of adipose progenitor and endothelial cells. Circ Res.

[32] Furuhata S, Ando K, Oki M, Aoki K, Ohnishi S, Aoyagi K (2007). Gene expression profiles of endothelial progenitor cells by oligonucleotide microarray analysis. Mol Cell Biochem.

[33] Ahmed TA, Dare EV, Hincke M (2008). Fibrin: a versatile scaffold for tissue engineering applications. Tissue Eng Part B Rev.

[34] Buchta C, Hedrich HC, Macher M, Höcker P, Redl H (2005). Biochemical characterization of autologous fibrin sealants produced by CryoSeal® and Vivostat® in comparison to the homologous fibrin sealant product Tissucol/Tisseel®. Biomaterials.

[35] Ratel D, Mihoubi S, Beaulieu E, Durocher Y, Rivard GE, Gingras D (2007). VEGF increases the fibrinolytic activity of endothelial cells within fibrin matrices: Involvement of VEGFR-2, tissue type plasminogen activator and matrix metalloproteinases. Thromb Res.

[36] Prager GW, Breuss JM, Steurer S, Mihaly J, Binder BR (2004). Vascular endothelial growth factor (VEGF) induces rapid prourokinase (pro-uPA) activation on the surface of endothelial cells. Blood.

[37] Rehman J, Traktuev D, Li J, Merfeld-Clauss S, Temm-Grove CJ, Bovenkerk JE (2004). Secretion of angiogenic and antiapoptotic factors by human adipose stromal cells. Circulation.

[38] Ehrbar M, Zeisberger SM, Raeber GP, Hubbell JA, Schnell C, Zisch AH (2008). The role of actively released fibrin-conjugated VEGF for VEGF receptor 2 gene activation and the enhancement of angiogenesis. Biomaterials.

[39] Kang HM, Kalnoski MH, Frederick M, Chandler WL (2005). The kinetics of plasmin inhibition by aprotinin in vivo. Thromb Res.

[40] Fergusson DA, Hébert PC, Mazer CD, Fremes S, MacAdams C, Murkin JM (2008). A comparison of aprotinin and lysine analogues in high-risk cardiac surgery. N Engl J Med.

[41] Shaw AD, Stafford-Smith M, White WD, Phillips-Bute B, Swaminathan M, Milano C (2008). The effect of aprotinin on outcome after coronary-artery bypass grafting. N Engl J Med.

[42] Stamou SC, Reames MK, Skipper E, Stiegel RM, Nussbaum M, Geller R (2009). Aprotinin in cardiac surgery patients: is the risk worth the benefit?. Eur J Cardiothoracic Surg.

[43] Lafleur MA, Handsley MM, Knäuper V, Murphy G, Edwards DR (2002). Endothelial tubulogenesis within fibrin gels specifically requires the activity of membrane-type-matrix metalloproteinases (MT-MMPs). J Cell Sci.

[44] Hotary KB, Yana I, Sabeh F, Li X-Y, Holmbeck K, Birkedal-Hansen H (2002). Matrix metalloproteinases (MMPs) regulate fibrin-invasive activity via MT1-MMP-dependent and -independent processes. J Exp Med.

[45] Hiraoka N, Allen E, Apel IJ, Gyetko MR, Weiss SJ (1998). Matrix metalloproteinases regulate neovascularization by acting as pericellular fibrinolysins. Cell.

[46] Zamolodchikov D, Strickland S (2012). Aβ delays fibrin clot lysis by altering fibrin structure and attenuating plasminogen binding to fibrin. Blood.

[47] Dallabrida SM, Falls LA, Farrell DH (2000). Factor XIIIa supports microvascular endothelial cell adhesion and inhibits capillary tube formation in fibrin. Blood.

[48] Sacchi V, Mittermayr R, Hartinger J, Martino MM, Lorentz KM, Wolbank S (2014). Long-lasting fibrin matrices ensure stable and functional angiogenesis by highly tunable, sustained delivery of recombinant VEGF164. Proc Natl Acad Sci.

[49] Zhang H, Zhou L, Zhang W (2014). Control of scaffold degradation in tissue engineering: a review. Tissue Eng Part B Rev.

[50] Marx G, Mou X (2002). Characterizing fibrin glue performance as modulated by heparin, aprotinin, and factor XIII. J Lab Clin Med.

[51] Koutsioumpa M, Hatziapostolou M, Mikelis C, Koolwijk P, Papadimitriou E (2009). Aprotinin stimulates angiogenesis and human endothelial cell migration through the growth factor pleiotrophin and its receptor protein tyrosine phosphatase beta/zeta. Eur J Pharmacol.

[52] Kachgal S, Putnam AJ (2011). Mesenchymal stem cells from adipose and bone marrow promote angiogenesis via distinct cytokine and protease expression mechanisms. Angiogenesis.

[53] Ghajar CM, Kachgal S, Kniazeva E, Mori H, Costes SV, George SC (2010). Mesenchymal cells stimulate capillary morphogenesis via distinct proteolytic mechanisms. Exp Cell Res.

[54] Zvonic S, Lefevre M, Kilroy G, Floyd ZE, DeLany JP, Kheterpal I (2006). Secretome of primary cultures of human adipose-derived stem cells: modulation of serpins by adipogenesis. Mol Cell Proteomics.

[55] Kapur SK, Katz AJ (2013). Review of the adipose derived stem cell secretome. Biochimie.

[56] Riis S, Stensballe A, Emmersen J, Pennisi CP, Birkelund S, Zachar V (2016). Mass spectrometry analysis of adipose-derived stem cells reveals a significant effect of hypoxia on pathways regulating extracellular matrix. Stem Cell Res Ther.

[57] Tratwal J, Mathiasen AB, Juhl M, Brorsen SK, Kastrup J, Ekblond A (2015). Influence of vascular endothelial growth factor stimulation and serum deprivation on gene activation patterns of human adipose tissue-derived stromal cells. Stem Cell Res Ther.

[58] Monsuur HN, Weijers EM, Niessen FB, Gefen A, Koolwijk P, Gibbs S (2016). Extensive characterization and comparison of endothelial cells derived from dermis and adipose tissue: potential use in tissue engineering. PLoS One.

