# Correction to: The role of fibrinolysis inhibition in engineered vascular networks derived from endothelial cells and adipose-derived stem cells

**DOI:** 10.1186/s13287-018-1001-3

**Published:** 2018-10-07

**Authors:** Severin Mühleder, Karoline Pill, Mira Schaupper, Krystyna Labuda, Eleni Priglinger, Pablo Hofbauer, Verena Charwat, Uwe Marx, Heinz Redl, Wolfgang Holnthoner

**Affiliations:** 1 Ludwig Boltzmann Institute for Experimental and Clinical Traumatology, AUVA Research Centre, Donaueschingenstrasse 13, A-1200 Vienna, Austria; 2 Austrian Cluster for Tissue Regeneration, Vienna, Austria; 3 Institute of Molecular Biotechnology of the Austrian Academy of Science (IMBA), Vienna Biocenter (VBC), Vienna, Austria; 4 Department of Biotechnology, University of Natural Resources and Life Sciences (BOKU), Vienna, Austria; 5 TissUse GmbH, Berlin, Germany; 6 Present address: Division of Plastic and Reconstructive Surgery, Department of Surgery, Medical University of Vienna, Vienna, Austria

## Correction

The original article [1] contains numerous value errors in the graphs in Fig. 2b regarding the markers describing the values for total tubule length and mean tubule length without aprotinin at 2.5 mg/ml concentration of fibrinogen. The corrected version of this figure can be viewed ahead.


