# Supplementary material for: The role of fibrinolysis inhibition in engineered vascular networks derived from endothelial cells and adipose-derived stem cells
Source: Stem Cell Res Ther. 2018 Feb 12;9:35. doi: 10.1186/s13287-017-0764-2 (PMC5809876; doi:10.1186/s13287-017-0764-2)
Supplement: Supplementary file 2 — Co-cultures of HUVEC and ASC can be maintained in culture up to 15 weeks. (A) HUVEC and ASC were embedded in fibrin matrices containing 2.5 mg/mL fibrinogen. (B) Co-culture fibrin matrices containing 20 mg/mL were maintained in culture for up to 15 weeks. All images are representative for fibrin matrices from three independent experiments. Aprotinin was used in all samples. Scale bar: 200 μm. (DOC 1344 kb) [file 13287_2017_764_MOESM2_ESM.doc]

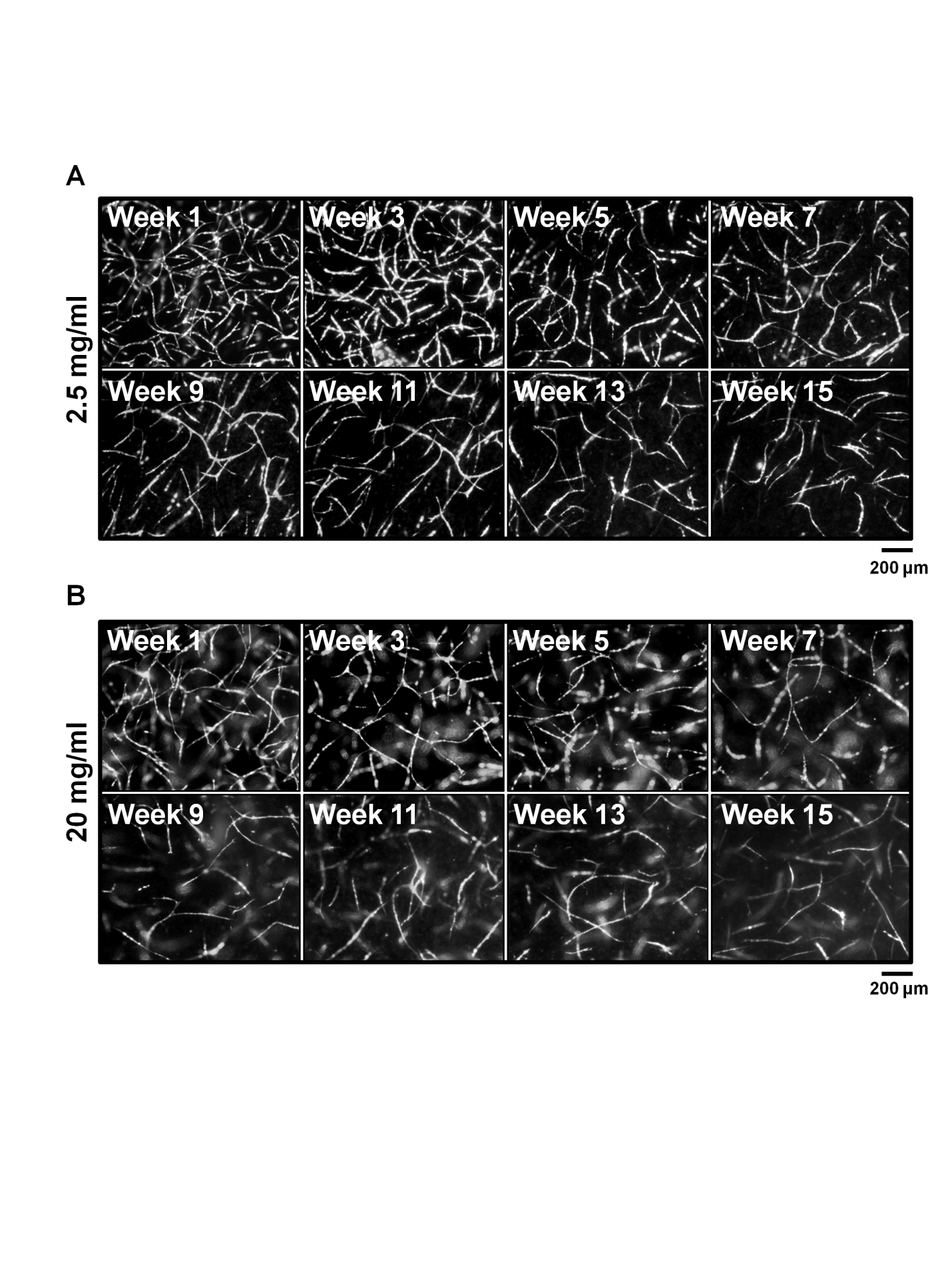


**Additional file 1: Co-cultures of HUVEC and ASC can be maintained in culture up to 15 weeks.** (A) HUVEC and ASC were embedded in fibrin matrices containing 2.5 mg/mL fibrinogen. (B) Co-culture fibrin matrices containing 20 mg/mL were maintained in culture for up to 15 weeks. All images are representative for fibrin matrices from three independent experiments. Aprotinin was used in all samples. Scale bar: 200 µm.
