# Supplementary material for: The role of fibrinolysis inhibition in engineered vascular networks derived from endothelial cells and adipose-derived stem cells
Source: Stem Cell Res Ther. 2018 Feb 12;9:35. doi: 10.1186/s13287-017-0764-2 (PMC5809876; doi:10.1186/s13287-017-0764-2)
Supplement: Supplementary file 3 — Influence of different aprotinin concentrations on HUVEC network formation. (A) Representative images of the effect of different aprotinin concentrations (0 KIU/ml, 5 KIU/ml, 10 KIU/ml, 20 KIU/ml, 30 KIU/ml and 100 KIU/ml) on HUVEC/ASC vascular network formation taken on day 28 of incubation. (B) Quantification of the network by number of junctions, tubules, total and mean tubule length. Increased aprotinin concentration results in a decreased number of tubules as well as junctions and total tubule length. Mean tubule length shows a dose-dependent increase, which peaks in samples with 20 KIU/ml aprotinin. Values are from two independent experiments using two different ASC donors; n = 2. Scale bar: 200 μm. (DOC 741 kb) [file 13287_2017_764_MOESM3_ESM.doc]

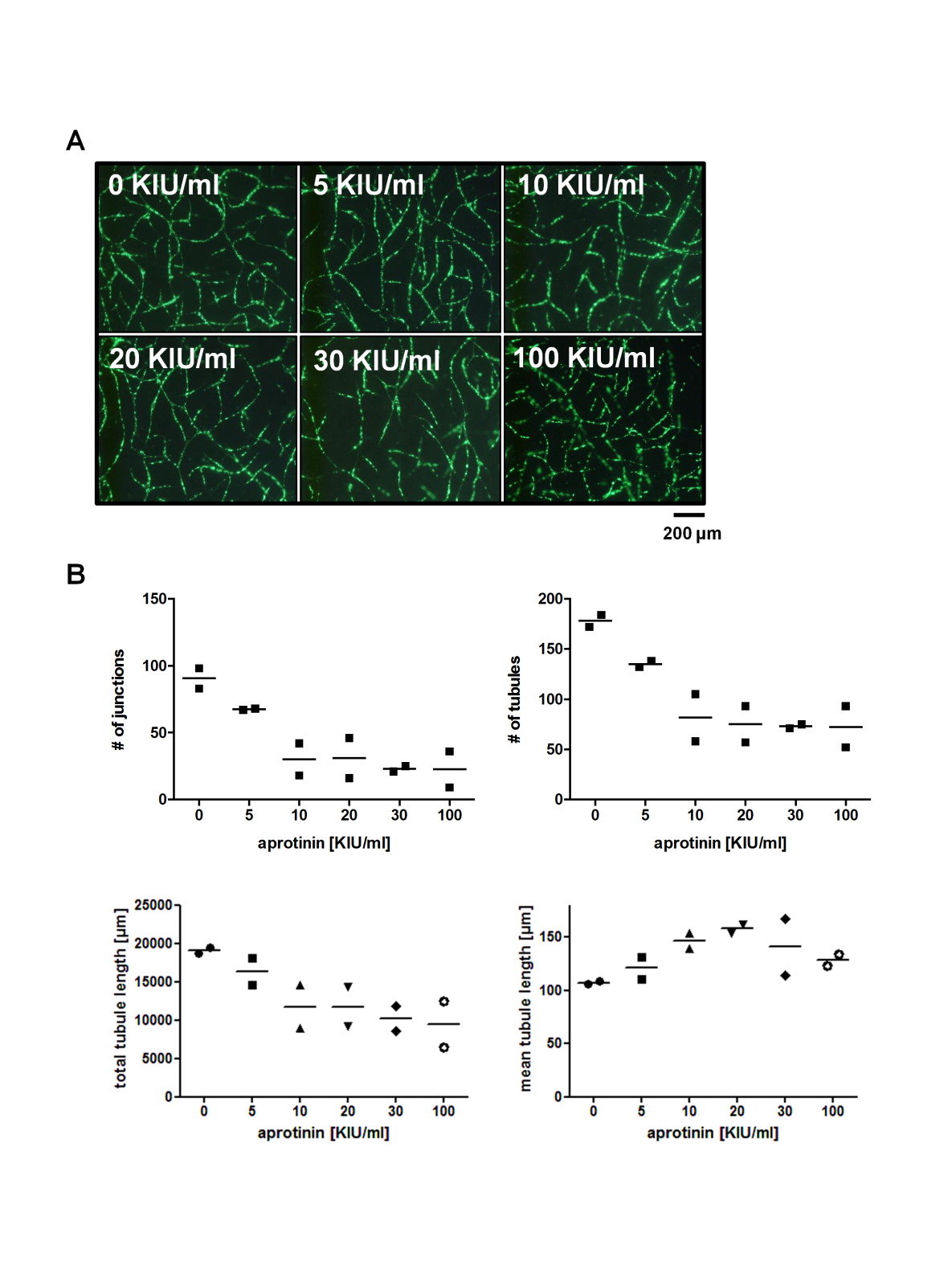


**Additional file 2: Influence of different aprotinin concentrations on HUVEC network formation.** (A) Representative images of the effect of different aprotinin concentrations (0 KIU/ml, 5 KIU/ml, 10 KIU/ml, 20 KIU/ml, 30 KIU/ml and 100 KIU/ml) on HUVEC / ASC vascular network formation taken on day 28 of incubation. (B) Quantification of the network by number of junctions, tubules, total and mean tubule length. Increased aprotinin concentration results in a decreased number of tubules as well as junctions and total tubule length. Mean tubule length shows a dose-dependent increase which peaks in samples with 20 KIU/ml aprotinin. Values are from two independent experiments using two different ASC donors; n = 2. Scale bar: 200 µm.
