# Supplementary material for: The role of fibrinolysis inhibition in engineered vascular networks derived from endothelial cells and adipose-derived stem cells
Source: Stem Cell Res Ther. 2018 Feb 12;9:35. doi: 10.1186/s13287-017-0764-2 (PMC5809876; doi:10.1186/s13287-017-0764-2)
Supplement: Supplementary file 4 — Aprotinin does not impair network formation of endothelial cells in a 2D setup. CD31 staining of endothelial cells (either HUVEC or ECFC) reveals tube-like structures when co-cultured with ASC in a 2D-setup devoid of fibrin. Aprotinin (100 KIU/ml) has no effect on HUVEC 2D tube formation. Scale bar: 100 μm or 50 μm as indicated. (DOC 370 kb) [file 13287_2017_764_MOESM4_ESM.doc]

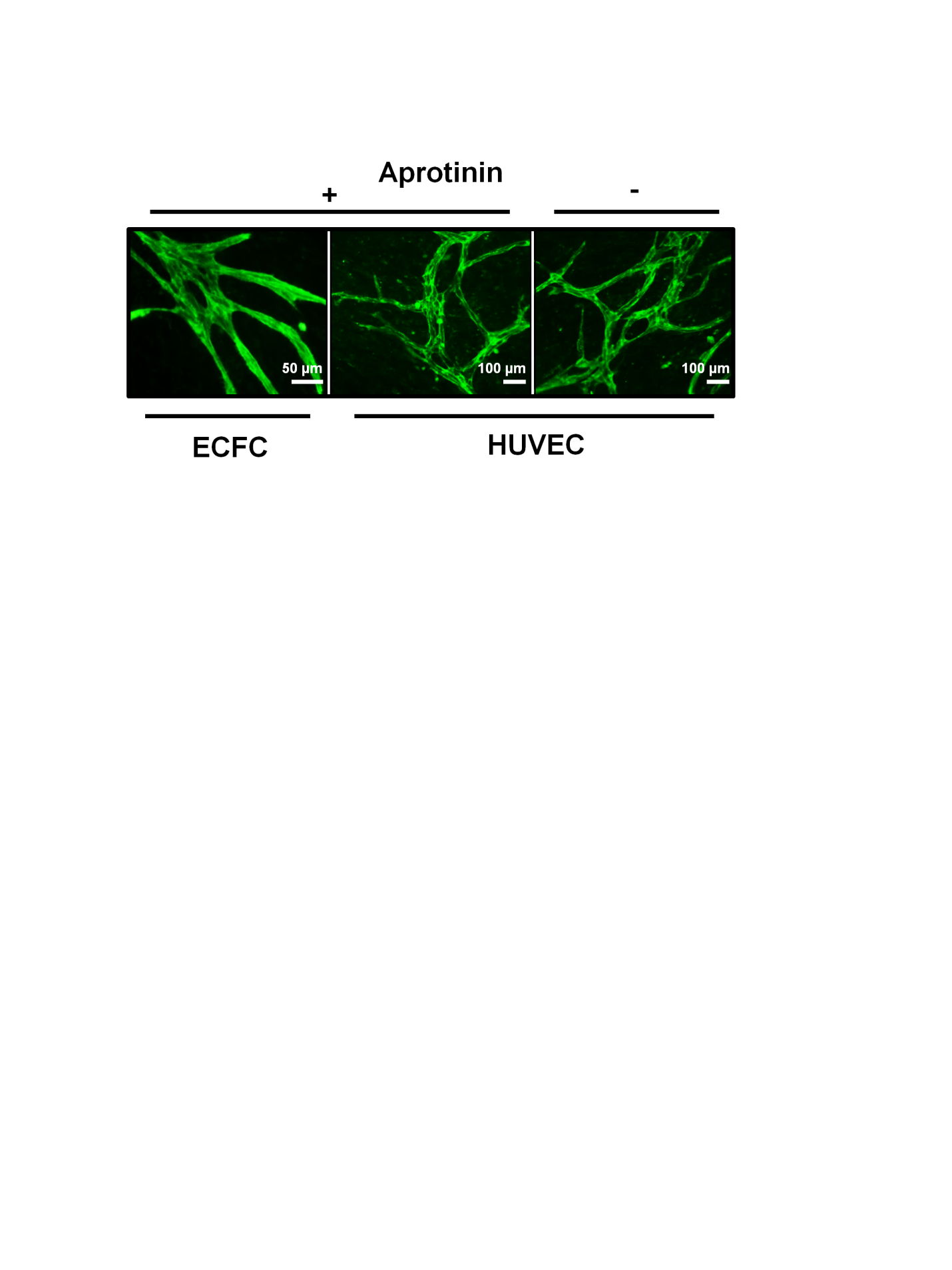


**Additional file 3**: **Aprotinin does not impair network formation of endothelial cells in a 2D setup.** CD31 staining of endothelial cells (either HUVEC or ECFC) reveals tube-like structures when co-cultured with ASC in a 2D-setup devoid of fibrin. Aprotinin (100 KIU/ml) has no effect on HUVEC 2D tube formation. Scale bar: 100 µm or 50 µm as indicated.
