# Supplementary material for: The role of fibrinolysis inhibition in engineered vascular networks derived from endothelial cells and adipose-derived stem cells
Source: Stem Cell Res Ther. 2018 Feb 12;9:35. doi: 10.1186/s13287-017-0764-2 (PMC5809876; doi:10.1186/s13287-017-0764-2)
Supplement: Supplementary file 5 — Thrombin transiently activates EC. HUVEC-ASC clots were fixed and immunofluorescence was performed against E-Selectin after 4 h, 1 day, 4 days and 7 days. Staining revealed transient activation of EC via thrombin after 4 h. TNF-α (10 ng/ml), serving as a positive control, activated EC more strongly after 4 h than thrombin alone. No aprotinin was used in any sample. n = 8 from two independent experiments. Scale bar: 200 μm. (DOC 322 kb) [file 13287_2017_764_MOESM5_ESM.doc]

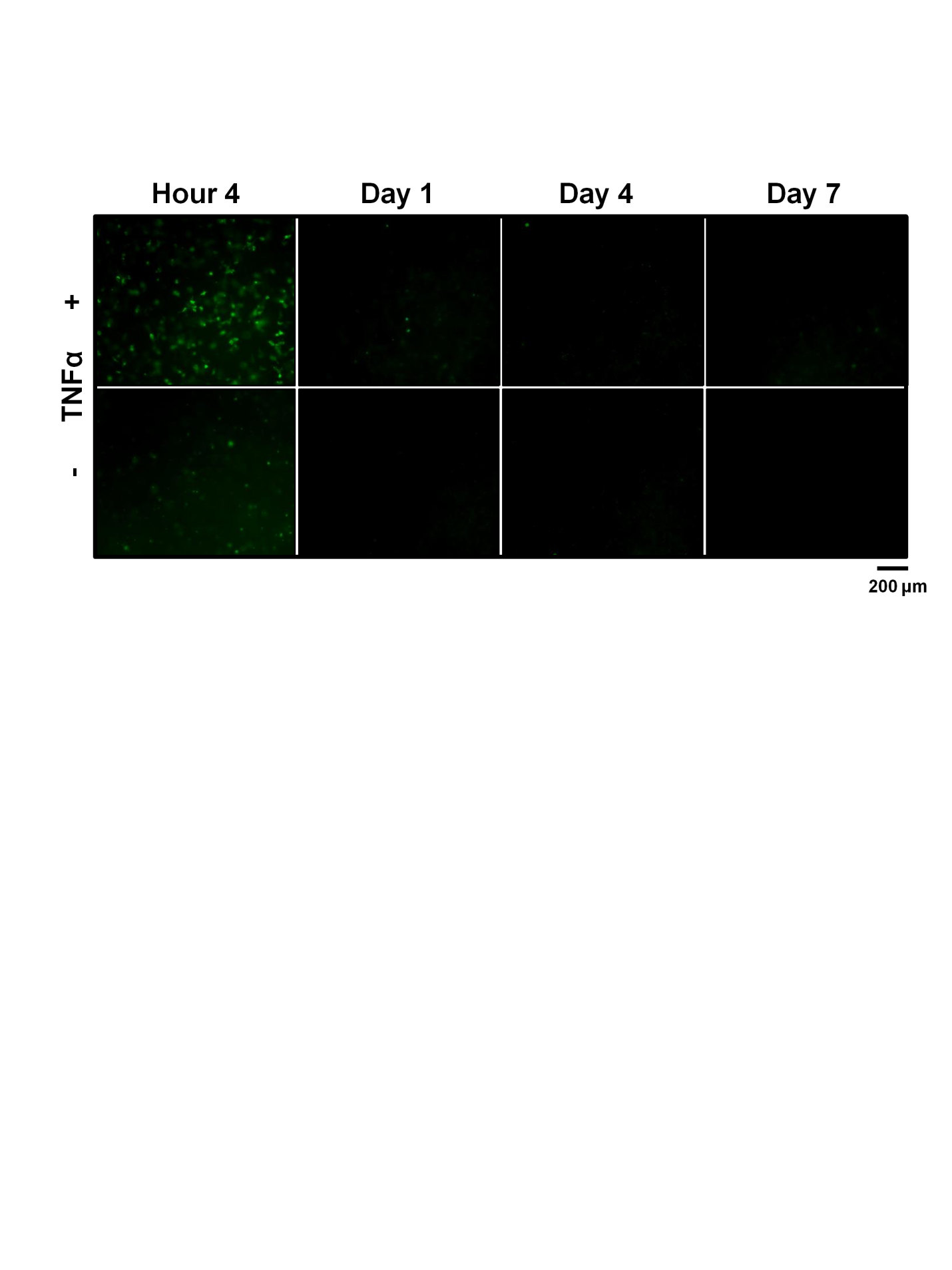


**Additional file 4: Thrombin transiently activates EC.** HUVEC-ASC clots were fixed and immunofluorescence was performed against E-Selectin after 4 h, 1 day, 4 days and 7 days. Staining revealed transient activation of EC via thrombin after 4 h. TNF-α (10 ng/ml), serving as a positive control, activated EC more strongly after 4 h than thrombin alone. No aprotinin was used in any sample. n = 8 from two independent experiments; Scale bar: 200 µm.
